# Hæmatology—II

**Published:** 1910-02-05

**Authors:** 


### Pathology.

### HÆMATOLOGY—II.

In our last article we had reached the description of the serum of the blood—*i.e.* the fluid in which the red corpuscles float. For ordinary practical purposes—for having the blood tested for Widal's reaction for typhoid or Malta fever—one takes the blood in capillary tubes, and then either by letting these stand or by centrifuging (quick method) separates the corpuscles from the serum. By filing through the tube the serum can be easily removed by a fine pipette or in the loop of a platinum needle, and so transferred to the slide, where, after being diluted, it can be mixed with suitable emulsions of the bacillus to be tested.

### Blood Pigments.

The pigments of the blood are three in number. (1) *Hæmoglobin.*—This gives the colour to the red blood corpuscles. It forms crystals which differ in shape in the different mammals, these being microscopic in size and of a bright red colour. In man they are of the shape of prismatic needles or rhombic plates, while in the squirrel, for example, they are hexagonal plates, and in the guinea-pig tetrahedral or octahedral masses. (2) *Hæmatin.*—This is the proper blood pigment, it being an amorphous dark brown or black material. It can be obtained in a crystalline form as hydrochlorate of hæmatin, the crystals being again microscopic in size, of a nut-brown colour, and having the shape of narrow rhombic plates. These have been termed hæmin crystals or Teichmann's crystals. (3) *Hæmatoidin.*—In extravasated human blood, blood crystals of a bright yellow or orange colour may be met with. They were called by Virchow, their discoverer, hæmatoidin. They are supposed to be identical with bilirubin obtained from human bile.

After having studied the various points previously mentioned, further methods of investigation still remain.

Tabulating these, they may be taken in the following order:—

1. The enumeration of the red and white blood corpuscles.
2. The estimation of the hæmoglobin.
3. The spectroscopic examination of the blood.
4. The determination of the coagulation time of the blood.
5. The estimation of the specific gravity of the blood.
6. The agglutination reaction.
7. The bacteriological examination of the blood.

### 1. The Enumeration of the Red and White Blood Corpuscles.

This is done by instruments called hæmocytometers, or in a rougher way the reds may be counted by an apparatus called the hæmatocrit (see later). The hæmocytometers in general use are Gower's, Thoma Leitz, or Thoma Zeiss, the last named being perhaps more generally used than the others and is the one recommended. The apparatus consists of a specially made slide with microscopic squares marked out on the circular central platform of glass. Surrounding this circle is a well, and outside this a square piece of glass. Each square is $\frac{1}{400}$ of a square millimetre, and the apparatus is so arranged that when the specially made cover glass is applied the depth of fluid between the cover glass and the bottom of any of the individual squares is $\frac{1}{10}$ of a millimetre. The cubic capacity of each square, therefore, will be $\frac{1}{10} \times \frac{1}{400} = \frac{1}{4000}$ c.mm. The squares are divided up into sets of sixteens, and as there are sixteen of these, that gives 256 squares. Dividing off these sets, however, are other squares with a line bisecting them into two, and if these are also considered there will be found to be 400 squares in all. In addition to the slide, the apparatus is also supplied with two pipettes, one for the reds, the other for the whites; each of these is graduated, has a bulb for mixing, and has certain figures on it. The former is marked .5 and 1 on the stalk, 101 above the bulb; the latter .5 and 1 on the stalk, 11 above the bulb. The reason of this is that the blood must be diluted before it can be conveniently counted, and diluting fluids have to be employed. For the reds a 10 per cent. solution of sodium sulphate is suitable, this preventing hæmolysis; for the whites, 1 per cent. glacial acetic acid tinted with methylene blue (*e.g.* glacial acetic, pure, 1; distilled water, 100 c.c.; methylene blue saturated watery solution, filtered, 15 to 20 drops) is suitable, the acetic acid dissolving the reds and leaving only the whites, which are faintly tinged with blue. Some prefer counting the reds and whites together, and if this plan is adopted Toisson's solution or some similar one which will preserve both is required. The formula of Toisson's fluid is as follows:—

| | | |
|---|---|---|
| ℞ | Methyl violet, 5 B. ... ... | 0.025 grm. |
| | Sod. chlorid. ... ... ... | 1.000 grm. |
| | Sod. sulph. ... ... ... | 8.000 grm. |
| | Neut. glycerin ... ... ... | 30.000 grm. |
| | Aq. distillat ... ... ... | 160.000 grm. |

Filter before using.

*Method of Procedure.*—Suck up blood to .5 or 1 in the red pipette; sod. sulph. sol. to 101, this giving a dilution of 1-200 or 1 in 100 as the case may be. (2) Suck up blood to .5 or 1 in the white pipette (former preferable because less blood required); acetic acid methylene blue solution to 11, this giving a dilution of 1-20 or 1-10. Shake after sucking up to be sure the blood is properly mixed; there is a small glass bead in the bulb for the purpose of facilitating thorough mixing. A drop of the mixture is then blown out on to the central platform (care being taken that it is not sufficiently big to overflow into well when cover glass is applied), the cover glass applied, and after standing for five minutes to let the corpuscles settle the cells may be counted. For the reds count 40 squares for a minimum; for the whites count the whole 400 squares (the more squares counted, of course, the more accurate the results).

*Calculation* (*Reds*).

$$\frac{\text{No. of corpuscles in 40 squares} = x}{40} \times 4{,}000 \text{ cubic capacity of square} \times 100 \text{ (dil.)} = \text{No. of r.b.c. per c.mm.}$$

*Example.*

$$\frac{375 \text{ (r.b.c. in 40 squares)}}{40} \times 4{,}000 \times 100 = 3{,}750{,}000 \text{ r.b.c. per c.mm.}$$

*Whites.*

$$\frac{\text{No. of whites in 400 squares}}{400} \times 4{,}000 \text{ cubic capacity} \times 10 \text{ (dil.)} = \text{No. of whites per c.mm.}$$

*Example.*

$$\frac{70 \text{ (w.b.c. in 400 squares)}}{400} \times 4{,}000 \times 10 \text{ (dil.)} = 7{,}000 \text{ w.b.c. per c.mm.}$$

*i.e.*, if 400 squares counted add on two nothings to result.

Wright has devised (to some people a much easier method) a method of counting the blood by fields, a description of which will be given in next article.

